# Ultrasound Elastography in Chronic Liver Disease: From Clinical Applications to Quality Assurance and Future Perspectives

**DOI:** 10.3390/diagnostics16152303

**Published:** 2026-07-23

**Authors:** Rute Santos, Raquel Reis

**Affiliations:** 1 Medical Imaging and Radiotherapy Department, Coimbra Health School, Polytechnic University of Coimbra, 3045-093 Coimbra, Portugal; 2 Polytechnic University of Coimbra, Rua da Misericórdia, Lagar dos Cortiços, S. Martinho do Bispo, 3045-093 Coimbra, Portugal; 3 H&TRC—Health & Technology Research Centre, Coimbra Health School, Polytechnic University of Coimbra, 3045-093 Coimbra, Portugal; 4 Interdisciplinary Centre for the Study of Human Performance, University of Coimbra, 3004-531 Coimbra, Portugal; 5 Clínica de Imagiologia Diagnóstica e de Intervenção, Hospital de Santo António, Unidade Local de Saúde de Santo António, 4099-001 Porto, Portugal; silvareisraquel@gmail.com

**Keywords:** ultrasound elastography, vibration-controlled transient elastography, shear wave elastography, liver fibrosis, chronic liver disease, hepatic imaging, non-invasive diagnosis, quality assurance

## Abstract

To provide a clinically oriented overview of ultrasound elastography in chronic liver disease, focusing on currently available techniques, their clinical applications, quality assurance, technical pitfalls, and emerging developments that may shape future liver imaging practice. A narrative review of the current literature was conducted using international guidelines, systematic reviews, and original studies retrieved from major scientific databases. Particular emphasis was placed on the clinical applications of ultrasound elastography, quality assurance procedures, interpretation of liver stiffness measurements, and future technological developments. Vibration-controlled transient elastography (VCTE) and shear wave elastography (SWE) have become established non-invasive techniques for liver fibrosis assessment, demonstrating excellent diagnostic performance, particularly for advanced fibrosis and cirrhosis. Beyond fibrosis staging, ultrasound elastography contributes to prognostic stratification, treatment planning, and longitudinal disease monitoring across a broad spectrum of chronic liver diseases. However, liver stiffness measurements may be influenced by technical and biological confounding factors, including inflammation, cholestasis, hepatic congestion, obesity, and postprandial status, highlighting the importance of standardised acquisition protocols and careful clinical interpretation. Emerging developments, including spleen stiffness assessment, multiparametric ultrasound, and artificial intelligence-assisted image analysis, are expected to further improve diagnostic accuracy and support precision hepatology. Ultrasound elastography has become an essential component of the non-invasive evaluation of chronic liver disease, substantially reducing the need for liver biopsy while improving fibrosis staging, prognostic stratification, and longitudinal patient monitoring. Its optimal clinical implementation requires appropriate patient selection, adherence to quality assurance procedures, and careful interpretation within the broader clinical context. Future advances in multiparametric ultrasound, artificial intelligence, and international standardisation are expected to further strengthen the role of ultrasound elastography within precision hepatology and personalised liver disease management. Ultrasound elastography should be integrated into routine liver imaging as part of a multimodal diagnostic approach. Standardised acquisition protocols, continuous quality assurance, and appropriate operator training are essential to ensure reliable and reproducible liver stiffness measurements. Radiographers play a key role in examination quality, protocol adherence, and multidisciplinary patient care, contributing to accurate diagnosis and improved clinical decision-making.

## 1. Introduction

Among the non-invasive diagnostic approaches currently available, ultrasound elastography has emerged as one of the most important advances in hepatology over the last two decades. By enabling quantitative assessment of liver stiffness, ultrasound elastography provides a reliable non-invasive surrogate marker of hepatic fibrosis and has substantially reduced the need for liver biopsy in many clinical scenarios. Vibration-controlled transient elastography (VCTE) and shear wave elastography (SWE) are now widely implemented in clinical practice and are recommended by international guidelines for the assessment, risk stratification, and longitudinal monitoring of patients with chronic liver diseases [1,2,3,4].

Despite their excellent diagnostic performance, particularly for advanced fibrosis and cirrhosis, several technical, methodological, and clinical challenges remain. Liver stiffness measurements are influenced by multiple technical and physiological factors, including obesity, hepatic inflammation, cholestasis, hepatic congestion, and postprandial changes [2,3,5,6,7,8]. In addition, differences between ultrasound platforms, limited inter-vendor standardisation, and the lack of universally accepted disease-specific cut-off values continue to restrict direct comparison of results across elastography techniques and manufacturers [2,3,5,9,10,11,12,13].

Recent technological developments, including spleen stiffness assessment, multiparametric ultrasound, and artificial intelligence-based approaches, are expanding the role of elastography beyond conventional fibrosis staging and moving towards more individualised risk stratification and precision hepatology [2,12,14,15].

Therefore, the purpose of this narrative review is not only to summarise current ultrasound elastography techniques and their clinical applications in chronic liver disease, but also to critically discuss quality assurance issues, technical limitations, current unmet needs, and future perspectives that may further shape the role of elastography in hepatology.

## 2. Methods

For this review, a literature search was conducted using the electronic databases PubMed, Scopus, and Web of Science. The search included articles published in English up to March 2026. The search strategy included combinations of the following keywords: “ultrasound elastography”, “transient elastography”, “vibration-controlled transient elastography”, “shear wave elastography”, “point shear wave elastography”, “2D shear wave elastography”, “liver fibrosis”, “hepatic imaging”, and “chronic liver disease”.

Studies were selected according to their relevance to hepatic elastography, methodological quality, clinical applicability, and contribution to current evidence. Priority was given to recent original studies, consensus statements, clinical guidelines, meta-analyses, and review articles addressing the diagnostic performance and clinical implementation of elastography techniques in liver disease.

Articles unrelated to hepatic applications, studies with limited clinical relevance, duplicate publications, and papers lacking sufficient methodological detail were excluded. The selected literature was analysed and organised thematically according to elastography modality and clinical context to provide a structured synthesis of current evidence.

As this was a narrative review, a formal systematic review protocol or meta-analysis methodology was not applied. Nevertheless, efforts were made to include representative, high-quality, and clinically relevant evidence in order to minimise selection bias.

## 3. Results

### 3.1. Ultrasound Elastography Techniques

Ultrasound elastography encompasses a group of imaging techniques that estimate tissue stiffness by measuring tissue deformation or shear wave propagation. Over the past two decades, elastography has evolved from qualitative strain imaging to highly standardised quantitative methods that are now incorporated into the routine assessment of chronic liver disease [1,3,12]. Currently, the most widely used techniques include strain elastography (SE), point shear wave elastography (pSWE), two-dimensional shear wave elastography (2D-SWE), and vibration-controlled transient elastography (VCTE).

Although all techniques aim to estimate liver stiffness as a surrogate marker of fibrosis, they differ substantially regarding acquisition methodology, imaging guidance, diagnostic performance, standardisation, and clinical applicability [1,2,3,12].

#### 3.1.1. Strain Elastography (SE)

Strain elastography is a qualitative or semi-quantitative technique that estimates tissue deformation following external compression or physiological motion. Owing to its simplicity, low cost, and wide availability, it has been extensively applied in the evaluation of superficial organs such as the breast, thyroid, prostate, and lymph nodes [1,12]. However, the technique remains highly operator-dependent, lacks standardised quantitative measurements, and demonstrates limited reproducibility between examinations and ultrasound systems [1,12]. Although strain elastography has demonstrated value in musculoskeletal and other superficial tissue applications, its limited reproducibility and lack of quantitative standardisation restrict its role in chronic liver disease [1,16].

Because liver fibrosis requires accurate and reproducible quantitative assessment, strain elastography has only a limited role in chronic liver disease and is generally considered unsuitable for routine fibrosis staging. Consequently, current international recommendations favour quantitative shear wave-based techniques for hepatic applications [1,3,12].

#### 3.1.2. Shear Wave Elastography (SWE)

Shear wave elastography provides quantitative assessment of tissue stiffness by measuring the propagation velocity of mechanically induced shear waves generated through acoustic radiation force impulses [1,12]. Compared with strain elastography, SWE offers improved reproducibility and reduced operator dependency, making it more suitable for liver fibrosis assessment [1,3,12].

##### Point Shear Wave Elastography (pSWE)

Point shear wave elastography measures liver stiffness within a single predefined region of interest under B-mode ultrasound guidance. Its integration into conventional ultrasound systems enables simultaneous morphological assessment and stiffness quantification during routine examinations [1,12]. Nevertheless, the relatively small sampling area and the absence of real-time elasticity mapping may limit its ability to evaluate heterogeneous liver parenchyma [1,12].

##### Two-Dimensional Shear Wave Elastography (2D-SWE)

Two-dimensional shear wave elastography extends the capabilities of pSWE by generating real-time colour-coded stiffness maps over a larger region of interest. This allows simultaneous anatomical visualisation and quantitative stiffness assessment, facilitating region selection and potentially improving the evaluation of heterogeneous liver disease [2]. Several studies have demonstrated excellent diagnostic performance for advanced fibrosis, cirrhosis, and portal hypertension [2,3].

Despite these advantages, 2D-SWE remains susceptible to motion artefacts, suboptimal acoustic windows, severe steatosis, obesity, and inter-vendor variability [2,3]. Differences in acquisition algorithms and proprietary software among manufacturers continue to limit direct comparison of liver stiffness values and contribute to the lack of universally accepted diagnostic thresholds [10,11].

#### 3.1.3. Vibration-Controlled Transient Elastography (VCTE)

Vibration-controlled transient elastography (FibroScan^®^) measures liver stiffness by analysing the propagation velocity of mechanically generated shear waves without conventional B-mode imaging guidance [1,2,12]. The technique is rapid, portable, standardised, and extensively validated, making it the reference ultrasound elastography method for non-invasive liver fibrosis assessment [3,17].

The main advantages of VCTE include its ease of use, short acquisition time, excellent reproducibility, and extensive clinical validation across a broad range of chronic liver diseases [3,17]. These characteristics have established VCTE as the preferred technique for large-scale fibrosis screening and longitudinal patient follow-up [1,3,12,17].

Nevertheless, VCTE also presents important limitations. The absence of real-time imaging guidance may reduce measurement reliability in patients with anatomical variations or poor acoustic windows. Performance may also be affected by obesity, ascites, hepatic inflammation, congestion, cholestasis, and postprandial physiological changes [1,2,5,12]. Furthermore, although VCTE demonstrates excellent diagnostic accuracy for advanced fibrosis and cirrhosis, its ability to discriminate intermediate fibrosis stages remains more limited [5,17].

Overall, VCTE remains the most extensively validated and standardised ultrasound elastography technique currently available [3]. However, rather than representing competing technologies, VCTE and shear wave elastography should be considered complementary approaches. VCTE offers excellent standardisation and is particularly suitable for fibrosis screening, whereas 2D-SWE provides real-time anatomical guidance and may offer additional advantages in patients requiring targeted assessment or presenting with heterogeneous liver involvement [12,18].

**Figure 1:**
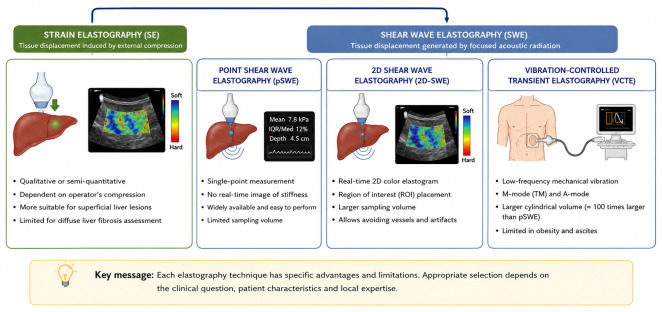
Classification and principles of ultrasound elastography techniques used in chronic liver disease. Adapted from the concepts presented in references [1,2,3,12].

The Figure 1 discribes ultrasound elastography techniques used in chronic liver disease.

A comparison of the principal characteristics, advantages, limitations, and quality criteria of the available ultrasound elastography techniques is presented in Table 1.

### 3.2. Clinical Applications in Chronic Liver Disease

Ultrasound elastography has become an integral component of the non-invasive evaluation of chronic liver disease. Current evidence supports its clinical use across a broad spectrum of chronic liver diseases for fibrosis staging, prognostic stratification, treatment planning, and longitudinal monitoring [1,3,12,17]. Although vibration-controlled transient elastography (VCTE) remains the most extensively validated technique, shear wave elastography (SWE) techniques also demonstrate excellent diagnostic performance for the assessment of liver fibrosis [1,2,3,12]. The choice of elastography modality should therefore be guided by the clinical indication, patient characteristics, equipment availability, and local expertise [1,3,12].

Although both VCTE and SWE techniques have demonstrated excellent diagnostic performance, most guideline-recommended liver stiffness thresholds and clinical decision algorithms have been validated using VCTE. Accordingly, unless otherwise specified, the cut-off values presented in the following sections refer to VCTE and should not be extrapolated directly to pSWE or 2D-SWE [1,2,3,12].

#### 3.2.1. Viral Hepatitis B and C

Ultrasound elastography plays a central role in the non-invasive assessment of chronic hepatitis B (HBV) and hepatitis C (HCV). In patients with HBV infection, liver stiffness measurement is particularly useful for identifying clinically significant fibrosis, distinguishing inactive HBV infection from active chronic hepatitis B, and supporting treatment decisions, prognostic stratification, and long-term patient monitoring [4,21,22]. Because liver stiffness may be influenced by necroinflammatory activity, particularly during hepatitis flares, elastography findings should always be interpreted together with biochemical parameters and the overall clinical context to avoid overestimation of fibrosis severity [4,5,22].

In HCV, the introduction of direct-acting antivirals has changed the role of elastography from treatment prioritisation towards long-term risk stratification. Although viral eradication is achieved in most patients, individuals with advanced fibrosis or cirrhosis continue to require surveillance for complications such as hepatocellular carcinoma and portal hypertension [17,21,23].

Overall, ultrasound elastography has become an essential component of fibrosis staging, risk stratification, and longitudinal follow-up in patients with chronic viral hepatitis [4,21,22].

#### 3.2.2. Metabolic Dysfunction-Associated Steatotic Liver Disease (MASLD)

Metabolic dysfunction-associated steatotic liver disease (MASLD) has become the leading indication for non-invasive liver fibrosis assessment owing to its rapidly increasing global prevalence and associated burden of advanced liver disease [4,24,25]. Ultrasound elastography is recommended as a first-line non-invasive technique for identifying patients at risk of advanced fibrosis while substantially reducing the need for liver biopsy [2,4,25,26]. Beyond fibrosis staging, liver stiffness measurement is increasingly used for prognostic stratification, longitudinal monitoring of disease progression, and assessment of response to lifestyle or pharmacological interventions [2,17,27].

#### 3.2.3. Autoimmune and Cholestatic Liver Diseases

In autoimmune hepatitis (AIH), primary biliary cholangitis (PBC), and primary sclerosing cholangitis (PSC), ultrasound elastography provides valuable information for fibrosis staging and prognostic assessment [28,29,30,31,32,33,34]. However, active inflammation and cholestasis may increase liver stiffness independently of fibrosis and should therefore be considered when interpreting results [31,32,34]. Following successful treatment, reductions in inflammatory activity are generally accompanied by lower liver stiffness values [31,34,35]. Consequently, ultrasound elastography should be considered a complementary tool that supports, rather than replaces, comprehensive clinical, biochemical, and imaging assessment [4,31,34].

#### 3.2.4. Special Clinical Situations

##### Liver Transplantation

Ultrasound elastography has become a valuable non-invasive tool for monitoring liver graft fibrosis following transplantation and may reduce the need for protocol biopsies in selected patients [22,36]. Vibration-controlled transient elastography (VCTE) has demonstrated good diagnostic performance for detecting recurrent or progressive graft fibrosis, particularly in recipients transplanted for chronic viral hepatitis and other chronic liver diseases [36].

Serial liver stiffness measurements may facilitate longitudinal graft surveillance, allowing earlier identification of patients who require further diagnostic evaluation or therapeutic intervention. However, elastography findings should always be interpreted in conjunction with clinical, biochemical, and imaging data, as postoperative changes, inflammation, cholestasis, vascular complications, and acute rejection may transiently increase liver stiffness independently of fibrosis [5,22,36].

Consequently, ultrasound elastography has become an important adjunct for the non-invasive follow-up of liver transplant recipients, although liver biopsy remains necessary when clinical, laboratory, and imaging findings are inconclusive [5,22,36].

##### Alcohol-Related Liver Disease

Ultrasound elastography demonstrates superior diagnostic performance compared with most serum biomarkers for the non-invasive assessment of advanced fibrosis in alcohol-related liver disease [22,37]. However, liver stiffness measurements may be substantially influenced by ongoing alcohol consumption, alcoholic hepatitis, and hepatic inflammation, potentially leading to overestimation of fibrosis severity [5,22,37].

Consequently, elastography should ideally be performed after a period of alcohol abstinence whenever clinically feasible, and results should always be interpreted together with biochemical parameters and the overall clinical context [37,38].

Serial liver stiffness measurements may also provide valuable information for monitoring disease progression and evaluating the response to alcohol withdrawal, as liver stiffness values frequently decrease following sustained abstinence, reflecting resolution of inflammation rather than regression of fibrosis alone [37,38].

##### Portal Hypertension

Portal hypertension is one of the most clinically relevant complications of advanced chronic liver disease and is closely associated with the development of oesophageal varices, ascites, hepatic decompensation, and increased mortality [4,39]. Although hepatic venous pressure gradient (HVPG) measurement remains the reference standard for diagnosing clinically significant portal hypertension (CSPH), its invasive nature limits its routine use in clinical practice [39].

In this context, liver stiffness measurement has become an important non-invasive tool for identifying patients at risk of CSPH. According to the Baveno VII consensus, CSPH can be ruled in patients with compensated advanced chronic liver disease when liver stiffness measurement by transient elastography is ≥25 kPa, while CSPH can be ruled out when liver stiffness is ≤15 kPa and platelet count is ≥150 × 10^9^/L [39].

Spleen stiffness measurement has emerged as a complementary biomarker that may improve non-invasive risk stratification, particularly in patients falling within the Baveno VII grey zone. Recent evidence suggests that combining liver stiffness, platelet count, and spleen stiffness may reduce the proportion of indeterminate cases and improve the identification of patients with CSPH or high-risk oesophageal varices [15].

Therefore, the combined assessment of liver and spleen stiffness may provide a more comprehensive non-invasive evaluation of portal hypertension than liver stiffness alone. However, technique-specific cut-off values, inter-vendor variability, and differences between VCTE and SWE platforms should be considered when interpreting results across different elastography platforms and clinical settings [2,12,31].

##### Congestion

Congestive hepatopathy may substantially increase liver stiffness in the absence of significant fibrosis because elevated central venous pressure and hepatic venous congestion increase tissue stiffness independently of chronic structural liver changes [5,22,32].

Consequently, elastography findings should always be interpreted within the broader clinical context, taking into account the patient’s cardiovascular status, biochemical parameters, and imaging findings to avoid overestimation of fibrosis severity [2,5,32]. In patients with heart failure, liver stiffness may decrease following optimisation of haemodynamic status, highlighting the dynamic nature of elastography measurements in congestive liver disease [5,22].

Therefore, ultrasound elastography should be considered a complementary diagnostic tool, with liver stiffness measurements interpreted alongside clinical and haemodynamic assessment rather than in isolation [2,5,22].

Representative liver stiffness cut-off values reported in different liver diseases are summarised in Table 2.

### 3.3. Clinical Integration and Interpretation of Ultrasound Elastography

Although vibration-controlled transient elastography (VCTE) remains the most extensively validated ultrasound elastography technique, no single modality is universally applicable across all clinical scenarios. VCTE is particularly suitable for large-scale fibrosis screening and longitudinal follow-up owing to its excellent standardisation, reproducibility, and extensive clinical validation [1,3,12,17]. In contrast, two-dimensional shear wave elastography (2D-SWE) offers the advantage of real-time B-mode guidance, enabling targeted assessment of heterogeneous liver parenchyma and improving region-of-interest selection during routine ultrasound examinations [2,3]. Recent WFUMB guidance further emphasises that VCTE and SWE should be considered complementary techniques rather than competing modalities, with the choice of technique tailored to the clinical indication, patient characteristics, equipment availability, and operator expertise [40]. Consequently, the choice of elastography technique should be individualised according to the clinical indication, patient characteristics, equipment availability, and operator expertise [12].

From a practical clinical perspective, VCTE is particularly advantageous for rapid, standardised liver fibrosis assessment and population-based chronic liver disease screening programmes, owing to its excellent reproducibility and extensive clinical validation. In contrast, 2D-SWE offers complementary advantages through real-time B-mode guidance, targeted evaluation of heterogeneous liver parenchyma, and seamless integration with conventional ultrasound examinations. Rather than representing competing technologies, VCTE and SWE should be regarded as complementary modalities, with the choice of technique tailored to the clinical indication, patient characteristics, equipment availability, and operator expertise [2,3,22].

Accurate interpretation of liver stiffness measurements requires integration with the patient’s clinical history, laboratory parameters, conventional ultrasound findings, and the underlying aetiology of liver disease, rather than relying on liver stiffness values alone [1,2,41]. Liver stiffness should always be interpreted in conjunction with potential confounding factors, including hepatic inflammation, cholestasis, congestion, recent food intake, obesity, and technical limitations that may influence measurement accuracy [1,3,5,20,32,42].

Quality assurance and operator competence are fundamental for obtaining reliable and reproducible liver stiffness measurements. Appropriate patient preparation, correct probe selection, adherence to quality criteria, and awareness of potential technical and physiological confounding factors are essential to minimise measurement variability and improve diagnostic accuracy [1,12,41]. Radiographers play a central role in ensuring protocol adherence, measurement consistency, and quality control throughout the examination, thereby supporting multidisciplinary clinical decision-making and contributing to high-quality patient care [1,3,12,41].

**Figure 2:**
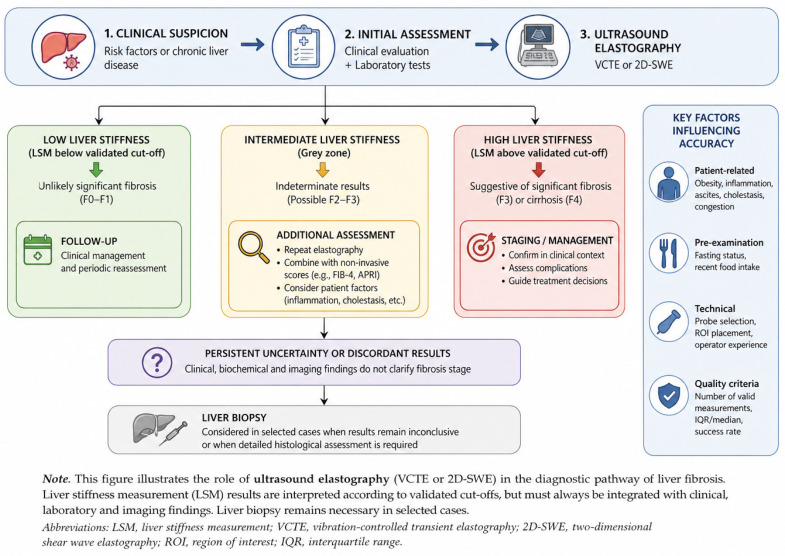
Clinical integration of ultrasound elastography in liver fibrosis assessment. The figure is based on current evidence and guidelines [1,2,3,4,12].

Overall, current evidence supports a multimodal approach in which ultrasound elastography complements, rather than replaces, clinical assessment, laboratory investigations, and conventional imaging. Appropriate selection of the elastography technique, combined with careful interpretation of liver stiffness measurements within the appropriate clinical context, is essential to maximise its diagnostic and prognostic value in patients with chronic liver disease [2,3,12]. This schematic representation summarises the role of ultrasound elastography (VCTE or 2D-SWE) in the diagnostic pathway of liver fibrosis (Figure 2). The suggested clinical use of ultrasound elastography for different clinical scenarios is summarized in Table 3.

**Table 3:** Suggested clinical use of ultrasound elastography according to clinical scenario.

Clinical Scenario	Preferred Technique	Rationale
Population screening for chronic liver disease	VCTE	Rapid, highly standardised, extensively validated, and suitable for large-scale fibrosis screening.
Routine abdominal ultrasound examination	pSWE	Easily integrated into conventional ultrasound systems, allowing simultaneous morphological assessment and liver stiffness measurement.
Assessment of heterogeneous liver parenchyma	2D-SWE	Real-time B-mode guidance enables accurate ROI placement and assessment of regional stiffness variations.
Obese patients	VCTE (XL probe) or 2D-SWE	The XL probe improves VCTE feasibility, whereas 2D-SWE may provide better anatomical guidance depending on the acoustic window.
Patients with ascites	2D-SWE	VCTE cannot be reliably performed because mechanical waves do not propagate through ascitic fluid.
Portal hypertension assessment	VCTE combined with spleen stiffness measurement	Improves non-invasive identification of clinically significant portal hypertension and risk stratification for oesophageal varices.
Longitudinal follow-up	VCTE	Excellent reproducibility and standardised acquisition facilitate serial liver stiffness measurements.
Indeterminate or discordant findings	Complementary use of VCTE and SWE, with liver biopsy when appropriate	Combining imaging findings with clinical assessment improves diagnostic confidence when non-invasive results are inconclusive.

Adapted and synthesised from international guidelines and review studies [2,3,4,12,22].

### 3.4. Quality Assurance and Technical Pitfalls

Reliable liver stiffness measurement depends not only on the elastography technique itself but also on appropriate patient preparation, standardised acquisition protocols, operator expertise, and correct interpretation of the results. Although ultrasound elastography has demonstrated excellent diagnostic performance for the assessment of advanced fibrosis and cirrhosis, inadequate acquisition technique and failure to recognise technical or physiological confounding factors may compromise measurement reliability and lead to inappropriate clinical interpretation [1,5,12,41].

#### 3.4.1. Patient Preparation and Examination Protocol

Appropriate patient preparation and correct examination technique are essential to minimise physiological variability and optimise measurement reliability [2,3,41]. Current recommendations advise that liver stiffness measurements should be performed after at least 3hours of fasting, as postprandial increases in hepatic blood flow may transiently elevate liver stiffness values independently of fibrosis [1,2,5,8,12]. Patients should be examined in the dorsal decubitus position with the right arm maximally abducted to widen the intercostal spaces and facilitate adequate access to the right hepatic lobe [2,3,41].

During VCTE examination, the probe should be positioned perpendicular to the skin surface through an intercostal approach, targeting homogeneous liver parenchyma while avoiding large vessels, biliary structures, focal lesions, and the liver capsule. Measurements should be obtained at least 1–2 cm below the liver capsule to minimise artefactual overestimation of liver stiffness and improve measurement reproducibility [2,3,41].

**Figure 3:**
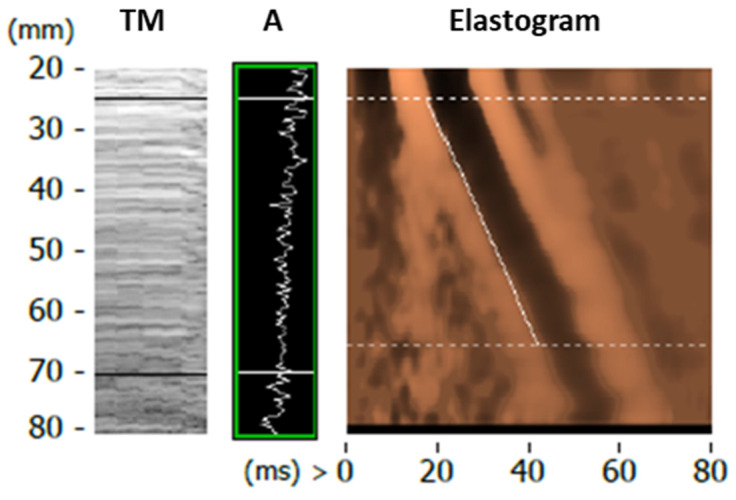
Optimal acquisition window identified by TM-mode and A-mode, resulting in Elastogram image, which is a 2D graphical representation of strain rates as a function of depth and time. (Images acquired by the authors).

**Figure 4:**
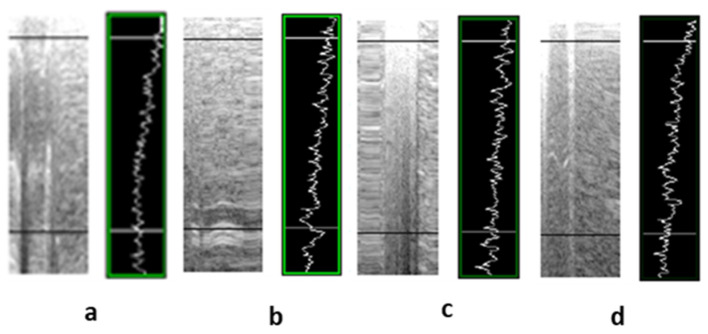
TM-mode window demonstrating a heterogeneous region of interest (**a**), vascular structures (**b**), image degraded by echo artefacts from bony structures (**c**) and reverberation artefact (**d**). (Images acquired by the authors).

An optimal acquisition window should be identified using Amplitude (A-mode) and Time Motion (TM-mode) displays, ensuring that the selected region of interest is homogeneous and free of vascular structures, rib shadowing, and interposed air (Figure 3 and Figure 4) [2,41].

#### 3.4.2. Quality Criteria

Strict adherence to quality criteria is fundamental to ensure reliable liver stiffness measurements. Current recommendations advise acquiring at least 10 valid measurements. Measurement reliability should be assessed using the interquartile range-to-median ratio (IQR/median), with values ≤30% [1,12,41].

Appropriate probe selection is equally important. The standard M probe is suitable for most patients, whereas the XL probe improves examination feasibility and reduces technical failure in patients with obesity [1,12]. The M probe operates at 3.5 MHz, measuring liver stiffness at 2.5–6.5 cm depth, while the XL probe operates at 2.5 MHz for depths of 3.5–7.5 cm and is indicated when the skin-to-liver capsule distance exceeds 2.5 cm. Probe selection is automatically determined by the system software based on this distance [1]. Although validated XL-specific thresholds for fibrosis staging are not yet established, its use improves the success rate of liver stiffness measurements, particularly in patients with BMI > 30 kg/m^2^ [43].

Operator experience also contributes significantly to measurement reproducibility. Standardised acquisition protocols, continuous training, and regular quality assurance programmes are therefore essential to minimise variability and optimise diagnostic performance [1,12,41].

Evidence from shear wave elastography studies in musculoskeletal imaging further highlights that tissue stiffness measurements are influenced by acquisition conditions and biomechanical factors, reinforcing the need for standardised protocols and careful interpretation across ultrasound elastography applications [44].

#### 3.4.3. Technical and Biological Confounding Factors

Liver stiffness measurements may be influenced by several conditions unrelated to fibrosis and should therefore always be interpreted cautiously. Active hepatic inflammation, particularly during acute hepatitis or alanine aminotransferase (ALT) flares, may substantially increase liver stiffness and lead to overestimation of fibrosis [5,7,31]. Similarly, extrahepatic cholestasis may elevate liver stiffness independently of fibrosis owing to increased biliary pressure [32].

Hepatic congestion associated with right-sided heart failure or other causes of venous congestion also increases liver stiffness and should be considered when interpreting elevated values [20]. Likewise, food intake transiently increases liver stiffness, reinforcing the importance of fasting before examination [5].

Technical factors, including obesity, narrow intercostal spaces, poor acoustic windows, respiratory motion, and inappropriate region-of-interest placement, may reduce examination feasibility or increase measurement variability. Awareness of these limitations is essential to avoid unreliable measurements and inappropriate clinical conclusions [1,12,41,44].

#### 3.4.4. Interpretation of Liver Stiffness Measurements

Liver stiffness measurements should always be interpreted cautiously, as several non-fibrotic conditions—including hepatic inflammation, cholestasis, congestion, obesity, and postprandial status—may increase liver stiffness independently of fibrosis [5,19,20,32].

Despite the major advances achieved with ultrasound elastography, liver biopsy remains necessary in selected clinical situations, particularly in patients with indeterminate liver stiffness values, discordant clinical findings, suspected mixed liver disease, or when inflammatory activity requires further histopathological characterisation [5,17]. Consequently, ultrasound elastography should be regarded as a complementary tool within a comprehensive diagnostic pathway rather than a complete replacement for histological assessment [4,5,22]. Several technical and physiological factors may influence liver stiffness measurements independently of fibrosis and should be considered during interpretation. These factors and their clinical implications are summarised in Table 4.

**Table 4:** Main factors influencing liver stiffness measurements and their clinical implications.

Factor	Potential Impact on Liver Stiffness Measurement	Clinical Implication
Postprandial state	Transient increase	Fast ≥ 3 h before examination
Hepatic inflammation	Increased liver stiffness	Interpret together with ALT and clinical findings
Cholestasis	Increased liver stiffness	Exclude biliary obstruction
Hepatic congestion	Increased liver stiffness	Consider underlying cardiac disease
Obesity	Increased technical failure and measurement variability	Use XL probe when appropriate and optimise acquisition
Hepatic steatosis	Variable influence	Interpret within the clinical context
Inter-vendor variability	Differences between elastography systems	Avoid direct comparison of measurements obtained with different platforms

Adapted and synthesised from previously published studies and international guidelines [3,5,17,19,20,32,41,42,43,45].

### 3.5. Current Challenges and Future Directions

Despite the remarkable advances achieved over the last two decades, several challenges continue to limit the widespread implementation and optimal clinical integration of ultrasound elastography. Although VCTE and shear wave elastography (SWE) have demonstrated excellent diagnostic performance for the assessment of advanced fibrosis and cirrhosis, important technical, methodological, and clinical challenges remain to be addressed [2,3,4].

One of the major challenges is the lack of complete standardisation across ultrasound platforms. Different manufacturers employ proprietary acquisition algorithms and processing software, resulting in variability in liver stiffness measurements and limiting direct comparison between systems. Consequently, universally applicable cut-off values remain difficult to establish, particularly for SWE techniques, highlighting the need for further harmonisation and international standardisation efforts [19].

Another important limitation concerns the assessment of intermediate fibrosis stages (F2–F3). Although elastography reliably identifies advanced fibrosis and cirrhosis, diagnostic performance decreases in intermediate stages, where liver stiffness values frequently overlap. Consequently, elastography findings should be interpreted within the broader clinical context, and liver biopsy continues to play an important role when non-invasive methods provide inconclusive or discordant results [5,17].

Recent developments are expanding the role of ultrasound elastography beyond conventional liver fibrosis assessment. Spleen stiffness measurement has emerged as a promising complementary biomarker for the non-invasive assessment of clinically significant portal hypertension and prediction of oesophageal varices, potentially improving risk stratification in patients with advanced chronic liver disease [1,3,12]. Similarly, multiparametric ultrasound approaches combining liver stiffness, attenuation imaging, shear wave dispersion, and conventional B-mode ultrasound are expected to provide a more comprehensive evaluation of hepatic disease than liver stiffness measurements alone [2,22,46].

Artificial intelligence and machine learning are also expected to play an increasingly important role in hepatic ultrasound. By integrating elastography findings with conventional ultrasound features, laboratory parameters, and clinical information, these technologies may improve diagnostic accuracy, reduce operator dependency, support automated quality control, and facilitate personalised risk prediction [12,14,47,48].

**Figure 5:**
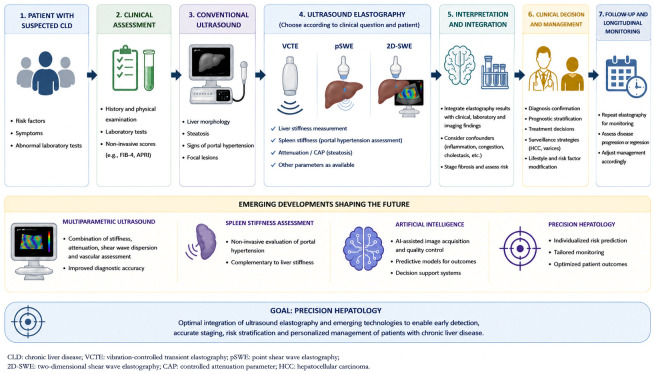
Clinical integration and future perspectives of ultrasound elastography in chronic liver disease. Adapted from references [1,12,41].

Future research should focus not only on further improving diagnostic accuracy but also on harmonising acquisition protocols, validating inter-vendor reproducibility, establishing disease-specific cut-off values, and evaluating the clinical impact of emerging technologies through prospective multicentre studies [2,3,12]. Such developments are expected to further strengthen the role of ultrasound elastography within precision hepatology and multidisciplinary patient care (Figure 5) [2,12,15].

## 4. Discussion

Ultrasound elastography has fundamentally transformed the non-invasive evaluation of chronic liver disease by providing reliable, reproducible, and widely accessible methods for liver fibrosis assessment. The progressive transition from liver biopsy towards non-invasive diagnostic pathways has substantially improved patient management by reducing procedure-related risks while allowing repeated assessment during disease monitoring [1,3,12,17].

Among the available techniques, VCTE remains the most extensively validated modality owing to its standardisation, reproducibility, and extensive clinical evidence. Nevertheless, advances in shear wave elastography have considerably expanded the diagnostic possibilities of conventional ultrasound by combining quantitative liver stiffness measurements with real-time anatomical guidance. Rather than representing competing technologies, VCTE and SWE should be regarded as complementary approaches whose selection should be guided by the clinical question, patient characteristics, equipment availability, and operator expertise [1,2,3,12].

An important finding emerging from the current literature is that diagnostic accuracy alone should no longer be considered the principal outcome when evaluating elastography techniques. Increasingly, the clinical value of ultrasound elastography lies in its integration into comprehensive diagnostic pathways, where liver stiffness measurements are interpreted together with clinical history, laboratory parameters, and conventional imaging findings. This multimodal approach improves diagnostic confidence, facilitates risk stratification, and supports personalised patient management while recognising that liver stiffness may be influenced by several physiological and pathological conditions unrelated to fibrosis [5,19,20,32].

Another important consideration is the growing shift from single-parameter assessment towards multiparametric ultrasound. The combination of liver stiffness measurements with attenuation imaging, shear wave dispersion, spleen stiffness assessment, and artificial intelligence-assisted image analysis has the potential to improve diagnostic performance beyond fibrosis staging alone. Although these technologies remain under continuous evaluation, they represent one of the most promising directions for the future development of precision hepatology [14,47,48].

From a practical perspective, the successful implementation of ultrasound elastography depends not only on technological advances but also on appropriate training, standardised acquisition protocols, and continuous quality assurance. Ensuring measurement reproducibility and recognising technical limitations remain essential prerequisites for reliable clinical decision-making. Consequently, multidisciplinary collaboration between radiologists, hepatologists, radiographers, and other healthcare professionals is fundamental to maximise the clinical benefits of ultrasound elastography [1,12,41].

Overall, current evidence supports the view that ultrasound elastography should no longer be regarded simply as an alternative to liver biopsy but rather as an integral component of contemporary chronic liver disease management [1,2,3,4].

## 5. Conclusions

Ultrasound elastography has become an essential component of the non-invasive evaluation of chronic liver disease, substantially reducing the need for liver biopsy while improving fibrosis staging, prognostic stratification, and longitudinal patient monitoring. Among the available techniques, VCTE remains the most extensively validated modality, whereas shear wave elastography offers complementary advantages through real-time anatomical guidance and integration with conventional ultrasound examinations.

Its optimal clinical implementation requires appropriate patient selection, rigorous quality assurance, and careful interpretation of liver stiffness measurements within the broader clinical context. Ultrasound elastography should therefore be regarded as a complementary tool that supports, rather than replaces, comprehensive clinical assessment and multidisciplinary decision-making.

Emerging developments, including multiparametric ultrasound, Controlled Attenuation Parameter (CAP), spleen stiffness assessment, and artificial intelligence-assisted image analysis, are expected to further enhance the diagnostic and prognostic capabilities of ultrasound elastography. To fully realise its clinical potential, future research should prioritise the harmonisation of acquisition protocols, validation of disease-specific cut-off values, inter-vendor standardisation, and prospective evaluation of emerging multiparametric and artificial intelligence-based approaches.

Ultimately, ultrasound elastography should be regarded not simply as an alternative to liver biopsy, but as an integral component of contemporary chronic liver disease management.

## 6. Key Clinical Messages

Ultrasound elastography has become a cornerstone of the non-invasive assessment of chronic liver disease, substantially reducing the need for liver biopsy.

Vibration-controlled transient elastography (VCTE) remains the most extensively validated technique for liver fibrosis assessment, while shear wave elastography provides complementary advantages through real-time anatomical guidance.

Liver stiffness measurements should always be interpreted together with clinical history, laboratory findings, and conventional imaging, as several non-fibrotic conditions may influence liver stiffness.

Appropriate patient preparation, standardised acquisition protocols, and quality assurance are essential to ensure reliable and reproducible measurements.

Emerging technologies, including multiparametric ultrasound, spleen stiffness assessment, and artificial intelligence, are expected to further improve non-invasive diagnosis and personalised management of chronic liver disease.

## Figures and Tables

**Table 1:** Comparison of ultrasound elastography techniques for liver fibrosis assessment.

Feature	Strain Elastography (SE)	Point Shear Wave Elastography (pSWE)	Two-Dimensional Shear Wave Elastography (2D-SWE)	Vibration-Controlled Transient Elastography (VCTE)
Physical principle	Tissue deformation after external or physiological compression	Acoustic radiation force impulse (ARFI) with point stiffness measurement	ARFI with real-time colour-coded stiffness mapping	External mechanical vibration generating shear waves
Quantitative assessment	No (qualitative/semi-quantitative)	Yes	Yes	Yes
B-mode imaging guidance	Yes	Yes	Yes	No
Main clinical application	Superficial organs (breast, thyroid, prostate)	Liver fibrosis assessment during conventional ultrasound	Liver fibrosis assessment and evaluation of heterogeneous liver parenchyma	Non-invasive liver fibrosis screening and longitudinal follow-up
Main strengths	Simple, inexpensive, widely available	Integrated into conventional ultrasound systems; quantitative assessment	Real-time stiffness mapping; larger sampling area; anatomical guidance	Highly standardised; rapid; portable; extensive clinical validation
Main limitations	Operator-dependent; poor reproducibility; non-quantitative	Small sampling area; no stiffness map	Motion artefacts; obesity; inter-vendor variability	No imaging guidance; reduced reliability in obesity and ascites
Level of Standardisation	Low	Moderate	Moderate	High
Quality criteria	No standardised criteria	Multiple valid measurements; homogeneous ROI; IQR/median < 30%	Homogeneous ROI; multiple measurements; IQR/median < 30%	≥10 valid measurements; IQR/median < 30%; fasting ≥ 3 h; appropriate probe selection

ROI, region of interest; IQR, interquartile range. Data summarised from published guidelines and review studies [1,3,5,12,17,19,20].

**Table 2:** Clinical interpretation of representative liver stiffness values across the major chronic liver diseases. Cut-off values vary according to ultrasound system, study population, and guideline recommendations and should always be interpreted within the appropriate clinical context [4,5,17,20].

Disease	Clinical Role of Elastography	Representative Liver Stiffness Cut-Off Values (kPa)	Important Considerations
Chronic hepatitis B	Fibrosis staging, treatment decision, and follow-up	Significant fibrosis (F2): ≈6.0–8.0 Advanced fibrosis (F3): ≈8.1–11.0 Cirrhosis (F4): ≈11.0–13.5	ALT flares and active necroinflammation may overestimate liver stiffness.
Chronic hepatitis C	Fibrosis staging before treatment and long-term surveillance	Significant fibrosis (F2): ≈7.0–8.5 Advanced fibrosis (F3): ≈9.5–12.5 Cirrhosis (F4): ≈12.5–14.5	Patients with advanced fibrosis require continued surveillance even after sustained virological response.
MASLD/MASH	Risk stratification, monitoring disease progression, and treatment response	F2: 6.6–7.8 F3: 7.1–10.4 F4: 10.3–22.3	Obesity and severe steatosis may reduce feasibility; XL probe may be required.
Primary biliary cholangitis (PBC)	Fibrosis staging and prognostic assessment	Advanced fibrosis: 9.6–10.7 Cirrhosis: 14.4–16.9	VCTE is the best validated non-invasive method in PBC.
Primary sclerosing cholangitis (PSC)	Fibrosis staging and prognosis	No universally accepted cut-off values	Exclude extrahepatic biliary obstruction before examination because cholestasis increases liver stiffness independently of fibrosis.
Autoimmune hepatitis (AIH)	Fibrosis staging and follow-up	No universally accepted cut-off values	Interpret together with ALT and inflammatory activity, particularly during active disease.
Alcohol-related liver disease	Fibrosis assessment and monitoring during abstinence	Cirrhosis: 11.5–25.8	AST, bilirubin, and ongoing alcohol intake influence liver stiffness measurements.

Legend: ALT, alanine aminotransferase; AST, aspartate aminotransferase; MASLD, metabolic dysfunction-associated steatotic liver disease; MASH, metabolic dysfunction-associated steatohepatitis; PBC, primary biliary cholangitis; PSC, primary sclerosing cholangitis; AIH, autoimmune hepatitis.

## Data Availability

No new data were created or analyzed in this study. Data sharing is not applicable to this article.

## Abbreviations

The following abbreviations are used in this manuscript:

SE	Strain Elastography
SWE	Shear Wave Elastography
pSWE	Point Shear Wave Elastography
2D-SWE	Two-Dimensional Shear Wave Elastography
VCTE	Vibration-Controlled Transient Elastography
LSM	Liver Stiffness Measurement
ROI	Region of Interest
IQR	Interquartile Range
ARFI	Acoustic Radiation Force Impulse
HBV	Hepatitis B Virus
HCV	Hepatitis C Virus
MASLD	Metabolic dysfunction-associated steatotic liver disease
MASH	Metabolic dysfunction-associated steatohepatitis
AIH	Autoimmune Hepatitis
PBC	Primary Biliary Cholangitis
PSC	Primary Sclerosing Cholangitis
CSPH	Clinically Significant Portal Hypertension
HVPG	Hepatic Venous Pressure Gradient
ALT	Alanine Aminotransferase
AST	Aspartate Aminotransferase
APRI	Aspartate Aminotransferase-to-Platelet Ratio Index
ELF	Enhanced Liver Fibrosis
EASL	European Association for the Study of the Liver
BMI	Body Mass Index
CAP	Controlled Attenuation Parameter
